# Circulating Nucleic Acid-Based Biomarkers of Type 2 Diabetes

**DOI:** 10.3390/ijms23010295

**Published:** 2021-12-28

**Authors:** Felipe Padilla-Martinez, Gladys Wojciechowska, Lukasz Szczerbinski, Adam Kretowski

**Affiliations:** 1 Clinical Research Centre, Medical University of Bialystok, 15276 Białystok, Poland; luis.padilla@umb.edu.pl (F.P.-M.); lukasz.szczerbinski@umb.edu.pl (L.S.); adam.kretowski@umb.edu.pl (A.K.); 2 Department of Endocrinology, Diabetology and Internal Medicine, Medical University of Bialystok, 15276 Białystok, Poland

**Keywords:** biomarkers, genomics, transcriptomics, epigenomics, type 2 diabetes, nucleic acid

## Abstract

Type 2 diabetes (T2D) is a deficiency in how the body regulates glucose. Uncontrolled T2D will result in chronic high blood sugar levels, eventually resulting in T2D complications. These complications, such as kidney, eye, and nerve damage, are even harder to treat. Identifying individuals at high risk of developing T2D and its complications is essential for early prevention and treatment. Numerous studies have been done to identify biomarkers for T2D diagnosis and prognosis. This review focuses on recent T2D biomarker studies based on circulating nucleic acids using different omics technologies: genomics, transcriptomics, and epigenomics. Omics studies have profiled biomarker candidates from blood, urine, and other non-invasive samples. Despite methodological differences, several candidate biomarkers were reported for the risk and diagnosis of T2D, the prognosis of T2D complications, and pharmacodynamics of T2D treatments. Future studies should be done to validate the findings in larger samples and blood-based biomarkers in non-invasive samples to support the realization of precision medicine for T2D.

## 1. Introduction

Type 2 diabetes (T2D) remains a significant clinical burden worldwide. Approximately 462 million individuals (6.28% of the world’s population) lived with T2D in 2017, and the prevalence continues to rise [1]. Better screening, diagnosis, and treatment approaches are needed to combat T2D. Early screening would ensure the timely implementation of lifestyle interventions for those at risk of developing T2D [2]. Early diagnosis is substantially beneficial for T2D patients. Even though T2D incidence peaks at age 55–59, many develop the disease at earlier ages and are often undiagnosed for years [1,3]. Early diagnosis would allow early treatment and prevent T2D complications, which are more challenging to treat [4]. Personalized T2D management, where drug choice is based on each patient’s characteristics, would maximize glycemic control efficiency and minimize side effects [5].

These goals can be achieved by identifying T2D biomarkers for each purpose. A biomarker is a biological observation that predicts an important endpoint or intermediate outcome in the clinical diagnosis [6]. The Biomarkers, EndpointS, and other Tools (BEST) glossary from the US National Institutes of Health (NIH) [7] defines seven biomarker categories: susceptibility/risk [8], diagnostic [9], monitoring [10], prognostic [11], predictive [12], pharmacodynamic/response [13], and safety [14] (Table 1).

Numerous studies have been conducted to identify T2D biomarkers. Several reviews have summarized genetic and non-genetic susceptibility/risk biomarkers for developing T2D [15,16,17]. Another review summarized omics-based biomarkers for diagnosing different types of diabetes and included non-invasive biomarkers from urine and saliva [18]. Research on omics-based and non-invasive T2D biomarkers has increased in recent years and proposed potential novel biomarkers. So, this review aims to summarize candidate human T2D biomarkers from a genomic, transcriptomic, and epigenomic perspective. We looked into T2D studies reporting all biomarkers categories, including risk and diagnostic biomarkers from blood, urine, and saliva samples. The gene names and symbols are listed in Table S1 (see Supplementary Materials).

## 2. Genomics Biomarkers for T2D

The science of genomes, or “genomics”, studies the structure, expression, and function of the whole DNA sequences of an organism. It has rapidly expanded towards a more functional level, studying the evolution, mapping, and editing of genomes [19]. Genomics analyzes genetic variants, such as single nucleotide polymorphisms (SNPs) and chromosomal abnormalities related to medical conditions [18]. T2D genomics studies used TaqMan qPCR assays, next-generation sequencing, and DNA microarray technologies.

### 2.1. Blood

Among all sample types, blood biomarkers represent the most accessible and most studied, given their properties and ease of collection in clinical practice [20]. A wide variety of T2D-related biomarkers in blood using genomic data has been reported (Table 2).

Heritability is a strong predictor of T2D (26–69% depending on the age of onset), thus motivating the search for genetic predictors of T2D [21]. One way to translate genetic data to a predictive measure of disease susceptibility is to add the risk effects of loci into a polygenic risk score (PRS) [22,23]. Prediction accuracy of a PRS is often assessed by measuring the area under the receiver operating characteristic curve (AUC). The AUC compares the rates of true positives and false positives, accounting for the overall performance of predictive models [24]. The first PRS for T2D was developed in 2006 using three genetic variants: *KCNJ11*, *PPARG*, and *TCF7L2* [25]. Two years later, three PRSs were developed using more SNPs. Two of them had 18 SNPs, and the third one had 11. Their AUC was 78%, 90%, and 74%, respectively [26,27,28]. In 2014, a 62-SNP PRS was developed using the DIAGRAMv3 Panel of Genes [29], with an AUC of 90% [30]. In 2016, Chikowore analyzed a South African population; a PRS using only four SNPs was created, taking into account sex, age, BMI, and systolic blood pressure as clinical risk factors, with an AUC of 66.5%. This research was the first to develop PRS in non-caucasian ethnic group with a high sensitivity and specificity [31]. Recent PRS models include many more SNPs, thanks to the larger genome-wide association studies (GWAS) in recent years. Furthermore, in 2016, Lall and colleagues published a PRS using 1000 SNPs from the DIAGRAM Panel of Genes, with an AUC of 77% [32].

Besides using SNPs in a PRS, some studies demonstrate other ways of utilizing SNPs as risk biomarkers. Ding and colleagues demonstrate that a low circulating level of sex hormone-binding globulin is a potential risk biomarker of T2D. Nevertheless, the clinical use of *SHBG* needs further examination [33]. In 2015, a study showed that endogenous bilirubin and the associated SNP, rs6742078 in the *UGT1A1* locus, are associated with the risk of T2D, making it a candidate as a risk biomarker [34]. Two years later, Wheeler identified 60 common genetic variants associated with HbA1c using genome-wide association meta-analyses from 82 European, African, East Asian, and South Asian ancestry cohorts. They found out that a G6PD deficiency can be clinically silent until illness strikes. Therefore, screening with direct glucose measurements in people with G6PD deficiency may be helpful as a risk predictor of T2D [35].

One of the first studies researching predictor biomarkers of T2D was done in 2010. In the study, the levels of plasma GAPDH, representing total cell-free DNA, were measured. The levels of cell-free GAPDH were significantly higher in the plasma samples of T2D diabetic patients, becoming a biomarker candidate [36]. The most recent study was done in 2019, where they assessed the potential role of mitochondria in T2D by analyzing blood-based indices of mitochondrial DNA copy number (mtDNACN) and cell-free mitochondrial DNA (CFmtDNA). As a result, they found a significant locus in the *LRRK2*/*MUC19* region, indicating that mitochondrial dysfunction is intimately linked to T2D prediction, therefore being a candidate biomarker [37].

**Table 2:** Genomics biomarkers from blood samples for T2D.

Sample Type	Profiling Method	Sample Size (Controls, T2D, Other)	Biomarker	Ref
Blood	Microarray	178, 178	*FTO*, *PSMD6*, *SLC44A3*, *C2CD4B*	[31]
Blood	GWAS, microarray	33,241 ^	*G6PD*	[35]
Blood	qRT-PCR	3669, 2409	*KCNK11*, *PPARG*, *TCF7L2*	[25]
Blood	qRT-PCR	23, 23, 6 **	*LRRK2*/*MUC19* (mtDNACN and CFmtDNA)	[37]
Blood	Microarray	2776 ^	*NOTCH2*, *BCL11A*, *THADA*, *IGF2BP2*, *PPARG*, *ADAMTS9*, *CDKAL1*, *VEGFA*, *JAZF1*, *SLC30A8*, *CDKNA/2B*, *HHEX*, *CDC123*, *TCF7L2*, *KCNJ11*, *INS*, *DCD*, *TSPAN8*	[27]
Blood	Microarray	9092, 1181	Panel of Genes DIAGRAM	[32]
Blood	qRT-PCR, DNA Sequencing	3471 ^	Panel of Genes DIAGRAMv3	[30]
Blood	qRT-PCR	2598, 2309	*TCF7L2*, *KCNJ11*, *CDKN2A*, *PPARG*, *ADAM30*, *CDKN2B*, *IGF2BP2*, *FTO*, *CDKAL1*, *SLC30A8*, *TSPAN8*, *CDC123*, *WFS1*, *TCF2*, *ADAMTS9*, *HHEX-IDE*, *THADA*, *JAZF1*	[26]
Blood	qRT-PCR	18,831 ^	*TCF7L2*, *PPARG*, *FTO*, *KCNJ11*, *NOTCH2*, *WFS1*, *CDKAL1*, *IGF2BP2*, *SLC30A8*, *JAZF1*, and *HHEX*	[28]
Blood	Microarray	3171, 210	*UGT1A1*	[34]
Blood Plasma	qRT-PCR	20, 25, 25 **	*GAPDH*	[36]
Blood Plasma	qRT-PCR	359, 359	*SHBG*	[33]

** T2D patients with complications or comorbidities; ^ cohort study.

### 2.2. Urine

Urine samples have several advantages above blood: the collection is easy and non-invasive, and the samples are available in large volumes. Anyone can collect urine samples, unlike blood samples which require clinical personnel [38]. Urine is not generally used for DNA analysis because the extracted DNA is less stable than blood. So, most T2D genomics studies collect blood and urine samples to study the associations between blood genetic variants and urine proteins (Table 3). However, recent studies have improved DNA extraction from urine [39]. One recent T2D study reported associations between mitochondrial DNA (mtDNA) from urine samples with T2D, demonstrating the possibility of urine-extracted DNA as biomarkers for T2D [40].

All T2D genomics urine studies looked into diabetic kidney disease (DKD), including diabetic nephropathy (DN) (Table 3). DKD is one of the significant complications of T2D and is the leading cause of end-stage renal failure. About one-third of diabetes patients will develop kidney disease, so identifying DKD biomarkers is essential for early prevention [43]. T2D studies on urinary biomarkers usually include patients with normo-, micro-, and macroalbuminuria.

Two studies found associations between SNPs measured from blood samples with urine proteins. A prospective study reported that rs10010131 in the *WFS1* gene is associated with a higher estimated glomerular filtration rate (eGFR) in T2D patients with increased albuminuria. This result suggests a potential role of *WFS1* as a risk biomarker of T2D and its kidney complications [41]. The second study examined the association between urine albumin and rs8192678 in the *PGC-1α* gene, a genotype previously related to nephropathy in T2D patients. The genotype was found to be associated with a 70% increase in urine albumin excretion in T2D patients with proteinuria compared to T2D patients with normoalbuminuria, making *PGC-1α* a candidate prognostic biomarker [42].

The only T2D study on urine-extracted DNA looked into mtDNA from healthy controls and T2D patients with and without proteinuria. Plasma and urine mtDNA content significantly differed between T2D patients and controls, where a reduction in plasma mtDNA content and increased urinary mtDNA/creatinine ratio were observed. The study did not report significant differences between T2D patients with and without proteinuria. So, the findings suggest that mtDNA could be a diagnostic biomarker of T2D [40].

### 2.3. Other Non-Invasive Biomarkers and the Use of Metagenomics

Saliva is another non-invasive sample to research biomarkers using genomic technology. Saliva-extracted DNA has comparable quantity and quality with blood, making saliva a suitable source of DNA for genetic studies. So, unlike urine, saliva has been used in large genetic epidemiological and metabolic studies [44]. However, there are no T2D studies that profile human DNA from saliva samples. One study profiled polymorphisms for *CHGA* from peripheral blood leukocytes and measured *CHGA* concentration in saliva (Table 4). The study found associations between two polymorphisms (rs9658635 in the promoter region and Glu264Asp in exon 6) with higher salivary *CHGA* production [45]. It remains to be seen whether these DNA biomarkers can also be detected in saliva samples.

In addition to human DNA, microbial DNA can also be extracted from saliva. Metagenomics studies the DNA sequences of an entire community of microorganisms [46]. The metagenomics studies aim to find associations between microbial profiles and human phenotypes. The technologies most frequently used in this area are 16S rRNA sequencing and whole-genome sequencing [18]. A recent study reported a higher relative abundance of *Bulleidia*, *Ruminococcaceae*, and *Helicobacter pylori* in T2D patients compared to healthy controls. However, the study was done in only nine T2D patients, so future studies are needed to confirm the findings [47] (Table 4).

**Table 4:** Genomics and metagenomics biomarkers from saliva and fecal samples for T2D.

Sample Type	Profiling Method	Sample Size (Controls, T2D, Other)	Biomarker	Ref
Fecal	16S rRNA sequencing	20, 20	Gut microbiome *(Ruminococcaceae*, *Lachnospiraceae*, and *Enterobacteriaceae*)	[48]
Fecal	16S rRNA sequencing	10, 10	Gut microbiome (*Akkermansia muciniphila*)	[49]
Fecal	16S rRNA sequencing	1427, 122, 1305 #	Gut microbiome (Bacterial sepecies with enriched ARG)	[50]
Fecal	16S rRNA sequencing	214, 48, 17 $, 151 *	Gut microbiome (*Escherichia*, *Veillonella*, *Blautia* and *Anaerostipes*)	[51]
Fecal	16S rRNA sequencing	55, 0, 71 #, 38 **	Gut microbiome (*Ruminococcus torques*)	[52]
Saliva	16S rRNA sequencing	27, 9, 31 #, 20 $, 46 **	Oral Microbiome (*Bulleidia*, *Ruminococcaceae*, and *Helicobacter pylori*)	[47]

* Pre-diabetes; ** T2D patients with complications or comorbidities; $ T2D patients with treatment; # non-T2D subjects with comorbidities.

T2D genetic studies have also been done using fecal samples. The genetic material from fecal samples is mostly bacterial, although human DNA can be detected in small amounts. There are efforts to improve human DNA extraction from stool samples, but significant challenges still exist for its use in population studies [53]. Therefore, all T2D studies with fecal samples are metagenomics studies (Table 4).

In 2016, a study showed evidence that the inflammation of the gut increased the values of biomarkers related to T2D. Microbial signatures of *Akkermansia muciniphila* demonstrate the existence of inflammation, increasing the risk of T2D before they are reflected by clinical markers [49]. Other metagenomics studies identified *Akkermansia muciniphila*, *Ruminococcus torques*, *Ruminococcaceae*, *Lachnospiraceae*, and *Enterobacteriaceae* to be associated with T2D [54,55,56,57]. In a critically high-risk population, a recent study demonstrated associations between *Escherichia*, *Veillonella*, *Blautia* and *Anaerostipes* with T2D [51]. Another study looked into the abundance of antibiotic resistance genes (ARG) in fecal microbiome profiles. The study found ARG enrichment in T2D patients, which could be potential biomarkers of T2D [50]. It remains to be seen if these T2D-associated changes in the gut contribute to T2D pathogenesis.

## 3. Transcriptomics Biomarkers of T2D

Transcriptomics is the comprehensive study of total gene expression levels in a cell or organism [58,59]. Most T2D messenger RNA (mRNA) research does not qualify this definition, as most studies profiled specific genes related to the study population. Nevertheless, the studies have identified potential mRNA biomarkers of T2D. The most common profiling method for T2D mRNA studies is targeted quantitative reverse-transcription PCR, where isolated RNAs are reverse transcribed to cDNA, then the expression levels of specific genes are measured quantitatively using qPCR. A few more recent T2D studies use microarray and RNA sequencing to measure many genes, but only in a small number of samples. Then, the top differentially expressed genes are validated using qRT-PCR in a larger number of samples. Currently, most T2D mRNA studies have been conducted with blood, but there is an increasing number of studies on non-invasive samples, such as urine and saliva.

### 3.1. Blood

Blood mRNA T2D studies have profiled gene expression in many sample types, from whole blood samples to leukocytes (Table 5). Only one study compared gene expression levels between two sample types: serum and urine [60]. There is little consensus on mRNA biomarkers because all studies selected genes related to their study population.

Blood mRNA studies on T2D patients without complications have identified elevated levels of inflammatory and senescence markers, as well as metabolically relevant receptor genes [61,62,63,64]. Plasma *NF-kB* expression levels were increased in pre-diabetic patients and further increased in T2D patients [62], suggesting that *NF-kB* can be a biomarker of early T2D diagnosis. In contrast, blood leptin mRNA levels were reduced in T2D patients compared to non-diabetic controls [65]. The discriminatory power of leptin mRNA was comparable to fasting blood glucose and HbA1c in distinguishing T2D patients and non-diabetic controls (AUROC = 0.95, 95% CI: 0.89–0.98) [65].

Studies on T2D patients with complications have focused on profiling genes related to T2D, inflammation, or specific diabetic complications [60,66,67,68,69,70,71]. For example, a study on diabetic kidney disease (DKD) patients selected *AEBP1* from a set of estimated glomerular filtration rate (eGFR)-correlated genes [66]. Another study on T2D patients with bone mass reduction or osteoporosis profiled *BSP* mRNA, a marker of bone metabolism [71]. The overall findings are increased inflammation and reduced expression of protective genes in patients with T2D complications.

One study identified a circulating biomarker for treatment response. A clinical trial administered placebo or zinc supplementation in overweight T2D patients and profiled *SOD1* expression in leukocytes [72]. The mRNA levels were significantly elevated in patients who received supplementation, compared to placebo and levels before treatment, and correlated with serum zinc concentrations. So, blood mRNAs can also be response biomarkers to evaluate treatment in T2D patients. More studies are needed to validate and identify other response biomarkers for T2D patients.

**Table 5:** Transcriptomics biomarkers from blood samples for T2D.

Sample Type	Profiling Method	Sample Size (Controls, T2D, Other)	Biomarker	Ref
Leukocytes	qRT-PCR	0, 35, 35 $	*SOD1*	[72]
Monocytes	qRT-PCR	30, 30, 30 **	*TLR2*, *TLR4*	[68]
PBMC	qRT-PCR	30, 30	*GLB1*, *p16*, *p21*, *p53*, *IL-6*, *TNF-a*, *SOCS-3*, *ERRγ*, *PPAR-γ*, *NOD-2*, *CYP2C9*	[61]
PBMC	qRT-PCR	20, 20	*TRAF-6*, *NF-kB*, *SOCS-3*	[64]
Plasma	qRT-PCR	30, 30, 30 *	*NF-kB*	[62]
Plasma	qRT-PCR	50, 55, 35 **	*Vaspin*	[69]
Plasma exosomes	qRT-PCR	10, 15, 15 **	*AEBP1*	[66]
Platelet	qRT-PCR	46, 43, 48 *, 36 **	*SFRP4*	[70]
Serum	qRT-PCR	45, 45, 45 #, 45 **	*BSP*	[71]
Serum	qRT-PCR	41, 33, 54 **	*TTP*, *IL-6*, *IL-8*	[60]
Serum exosome	qRT-PCR	0, 20, 24 **	*VEGF*	[67]
Whole blood	qRT-PCR	110, 148	*IL-23*, *TNF-a*, *IFN-g*	[63]
Whole blood	qRT-PCR	32, 71	*Leptin*	[65]

* Pre-diabetes; ** T2D patients with complications or comorbidities; $ T2D patients with treatment; # non-T2D subjects with comorbidities.

### 3.2. Urine

Similar to genetics studies, urinary mRNA studies have compared T2D patients with and without DKD (Table 6). The first study looked into the expression levels of *TTP* and inflammatory markers *IL-6* and *IL-18*. *TTP* encodes tristetraprolin, which is an anti-inflammatory protein. A significantly reduced *TTP* expression level was found in T2D patients, and the levels are further reduced in patients with macroalbuminuria. In contrast, *IL-6* and *IL-18* were significantly elevated in T2D patients, especially those with complications [60].

Another study looked into podocyte-associated markers: *NPHS1*, *NPHS2*, and *PODXL* [73]. Renal podocyte injury is pathologically significant in DN progression, so podocyte-associated genes might serve as monitoring biomarkers to evaluate kidney health. Urinary podocyte marker levels were significantly elevated in all T2D patients, including those with complications. Patients with macroalbuminuria had higher podocyte marker levels than normo- and microalbuminuria patients. These markers distinguished microalbuminuria patients from those without complications in univariate analyses, with AUCs of 0.685–0.961 [73].

A study with 242 participants used the presence of albuminuria and eGFR to define their study groups [74]. This study evaluated gene expression in urinary exosomes using RNA sequencing and validated eight mRNAs (*UMOD*, *SLC12A1*, *NDUFB2*, *OAZ1*, *PPARGC1A*, *NFE2L2*, *CD24*, *SMAD1*) using qRT-PCR. So, this is the first agnostic urinary mRNA study where markers were not selected from previously available knowledge. Among the eight mRNAs, *UMOD* and *SLC12A1* levels were elevated in T2D patients with complications compared to those without complications and healthy controls. Significant increases were also seen in *NDUFB2* and *OAZ1* in T2D patients without complications compared to healthy controls. The four mRNAs were significantly correlated to HbA1c%, but only *OAZ1* was correlated to T2D duration. A multi-gene signature was also developed using the eight mRNAs and linear discriminant analysis with cross-validation. The classifier distinguished severe DKD patients from T2D with an AUC of 0.90 [74].

**Table 6:** Transcriptomics biomarkers from urine samples for T2D.

Sample Type	Profiling Method	Sample Size (Controls, T2D, Other)	Biomarker	Ref
Urinary exosomes	RNA sequencing and qRT-PCR	41, 33, 54 **	*TTP*, *IL-6*, *IL-8*	[60]
Urine	qRT-PCR	20, 20, 40 **	*NPHS1*, *NPHS2*, *PODXL*	[73]
Urine	qRT-PCR	18, 29 #, 166, 34 **	*UMOD*, *SLC12A1*, *NDUFB2*, *OAZ1*	[74]

** T2D patients with complications or comorbidities; # non-T2D subjects with comorbidities.

### 3.3. Other Non-Invasive Biomarkers

Unlike genetic T2D biomarkers, there is limited research for mRNA biomarkers from saliva samples. One study reported differential salivary gene expression from 13 T2D patients: elevated *KRAS*, *SAT1*, *SLC13A2*, and *TMEM72* and reduced *EGFR* and *PSMB2* gene expressions [75]. The six biomarkers were first identified through microarray, then validated in 13 T2D patients and 13 healthy controls. A logistic model with four out of the six salivary biomarkers (*KRAS*, *SAT1*, *EGRF*, and *PSMB2*) discriminated T2D patients from healthy controls (AUC: 0.917, 95% CI: 0.809–1.000). So, salivary mRNAs are promising T2D biomarkers, but more studies on these samples are needed.

## 4. Epigenomics Biomarkers for T2D

Epigenomics studies the whole gene regulation of an organism or cell, including DNA methylation and microRNAs (miRNAs) [59,76]. Epigenetic factors regulate gene expression without modifying DNA sequence. For example, DNA methylation of CpG islands influences transcription factor binding, resulting in stable silencing of gene transcription [77]. Gene expression can also be regulated post-transcriptionally through miRNAs, which bind to target mRNAs and typically lead to translational repression [78]. The miRNA-mRNA interactions can be binary off-switch interactions or tuning interactions in response to an environmental cue [79].

There is growing interest in identifying circulating epigenetic biomarkers for T2D, especially since T2D has a substantial environmental influence, such as sedentary lifestyle and obesity. Identifying risk, predictive, monitoring, and response biomarkers are among the most common research aims in T2D epigenomic studies.

### 4.1. Blood

#### 4.1.1. DNA Methylation

All of the T2D DNA methylation blood studies were done on peripheral blood. The bisulfite conversion technologies vary; the most used were: EZ-DNA Methylation Kit from Zymo Research and EpiTect Bisulfite kit from QIAGEN. The DNA methylation technology most used in the studies was Illumina Human-Methylation450 BeadChip, followed by Qiagen PyroMark Pyrosequencing.

The study designs are different; nevertheless, there are CpG sites in specific genes repeated in several DNA methylation studies (Table 7). The T2D biomarkers most studied are CpG sites in the genes *ABCG1*, *TXNIP*, *SREBF1*, and *PHOSPHO1*. With six [80,81,82,83,84,85], four [80,81,84,86], three [80,84,85], and two [80,83] studies, respectively. The four methylation sites in those genes are in relevant pathways related to T2D. The gene *ABCG1* is involved in cholesterol and phospholipid transport and insulin secretion. DNA methylation at *ABCG1* has been associated with fasting insulin and HOMA-IR [87,88]. The gene *TXNIP* is a component of pancreatic β-cell biology, energy metabolism, and cellular redox regulation. *TXNIP* downregulation protects against obesity-induced diabetes by preventing β-cell apoptosis and preserving β-cell mass [89,90]. The gene *SREBF1* is activated by insulin and contributes to dyslipidemia and hepatic steatosis that occurs in obesity and T2D [91]. The gene *PHOSPHO1* is considered to be an attractive target for cardiovascular therapy. It has been found that DNA methylation at this gene is correlated positively with HDL levels [83,92]. The results of these studies open the way for functional studies to define their use as biomarkers of T2D and other metabolic comorbidities.

There are other DNA methylation studies of CpG sites that were reported. There were five publications during the 2010–2013 period, the first one discovered three CpG sites in the gene *TFAM* [93], the second found one CpG in the gene *FTO* [94], the third found one CpG site in the gene *IGFBP-1* [95], the fourth found seven CpG sites in the gene *PRKCZ* [96], and the fifth found thirteen CpG sites in the gene *TCF7L2* [97]. In 2014, an interesting study showed that lower *LINE-1* levels after global DNA methylation were associated with a higher risk of a poor carbohydrate metabolic status. This biomarker could be considered a risk factor of T2D and related metabolic disorders, independent of other established risk factors, therefore having a potential role as a possible biomarker [98]. From 2015 to 2017, another four studies suggested new CpG sites of possible interest for T2D biomarker development. The first one suggested five CpG sites in the gene *SLC30A8* [99], the second, one CpG site in the gene *TXNIP* [100], the third one also one CpG in the gene *TXNIP* [101], and the fourth one two CpG sites, one in the *MSI2* gene, and the other one in the *CXXC4* gene [102]. At the end of 2017, Van Otterdijk and colleagues reported a tendency between *PEG3* DNA methylation levels in peripheral blood leukocytes in T2D patients, and four CpG loci were differentially methylated between groups of participants. As a result, these loci might serve as biomarkers of T2D, although additional research is required to strengthen these observations further [103]. Previous studies had indicated that the *ELOVL5* expression is associated with T2D [104,105]. In 2018, Hwang et al. identified the *ELOVL5* gene as a novel epigenetic mark in an epigenome-wide analysis of the blood DNA methylation using T2D-discordant monozygotic twins. The results implied that epigenetic alterations in *ELOVL5* demonstrate DNA methylation and RNA expression changes. These provide insights into epigenetic biomarkers for T2D [106].

These methylation markers’ risk or diagnostic value may be small to justify them as a novel biomarker. Thus, further improvement should include CpG sites showing a higher methylation difference.

**Table 7:** DNA methylation biomarkers from blood samples for T2D.

Sample Type	Profiling Method	Sample Size (Controls, T2D, Other)	Biomarker	Ref
Peripheral blood leukocytes	Pyrosequencing	11, 25	4 CpG sites (*PEG3*)	[103]
Whole Blood	EpiTYPER assay	93,93	13 CpG sites (*TCF7L2*)	[97]
Whole Blood	Methylation-specific polymerase chain reaction (MSPCR)	45, 77	3 CpG sites (*TFAM*)	[93]
Whole Blood	Pyrosequencing	441, 509	5 CpG sites (*SLC30A8*)	[99]
Whole Blood	Microarray	120, 152	7 CpG sites (*PRKCZ*)	[96]
Whole Blood	Microarray	93, 30	*ABCG1* and *CCDC57*	[82]
Whole Blood	Microarray	6760, 306	*ABCG1*, *PHOSPHO1*, *SOCS3*, *SREBF1*, and *TXNIP*	[80]
Whole Blood	Microarray	11927, 1608	*ABCG1*, *PHOSPHO1*, *SOCS3*, *SREBF1*, and *TXNIP*	[80]
Whole Blood	Microarray	129, 129	cg06500161 (*ABCG1*) and cg02650017 (*PHOSPHO1*)	[83]
Whole Blood	DNA sequencing	176, 100	cg06500161 (*ABCG1*) and cg11024682 (*SREBF1*)	[85]
Whole Blood	Microarray	11,11	cg18681426 (*ELOVL5*)	[106]
Whole Blood	Microarray	835, 153	cg19693031 (*TXNIP*)	[100]
Whole Blood	Microarray	204, 151	cg19693031 (*TXNIP*)	[101]
Whole Blood	Microarray	98, 100	CpGs (in *ABCG1*, *LOXL2*, *TXNIP*, *SLC1A5*, and *SREBF1*)	[84]
Whole Blood	Pyrosequencing	606, 710	*FTO*	[94]
Whole Blood	Pyrosequencing	100, 240	*IGFBP-1* and *IGFBP-7*	[95]
Whole Blood	Pyrosequencing	100, 100	Long Interspersed Nucleotide Element 1 (*LINE-1*)	[98]
Whole Blood	Microarray	220, 220	*MSI2* and *CXXC4*	[102]
Whole Blood	Microarray	457, 256	*TXNIP* (cg19693031), *C7orf50* (cg04816311), *CPT1A* (cg00574958), and *TPM4* (cg07988171)	[86]
Whole Blood	Microarray	676, 174	*TXNIP*, *ABCG1* and *SAMD12*	[81]

#### 4.1.2. microRNA

Most T2D miRNA studies were done on blood samples, predominantly plasma, serum, or exosomes (Table 8). A few studies used whole blood, PBMCs, or endothelial progenitor cells. Almost all studies used qRT-PCR: profiling a large number of miRNAs using PCR panels, targeting a small number of miRNAs, or validating results from microarray, RNA sequencing, or nanostring. Some studies used RNA sequencing without qRT-PCR validation.

Despite the different study designs, there are commonly reported miRNAs. The most studied and promising T2D biomarker is hsa-miR-126. This miRNA is highly enriched in endothelial cells and is important for angiogenesis and wound repair [107]. It is down-regulated in T2D patients compared to non-diabetic controls and those with impaired glucose tolerance [62,107,108,109,110,111,112,113,114,115,116,117,118,119,120,121,122]. It is also further down-regulated in T2D patients with complications [111,115,119] and increases after six months of treatment (insulin with diet control and exercise) [116]. However, aspirin can affect the plasma hsa-miR-126 profile [123]. So, careful participant selection and interpretation of results are needed for future studies on hsa-miR-126.

Other miRNAs that were reported in at least four studies are hsa-miR-122-5p [120,124,125,126], hsa-miR-146a [64,120,127,128,129,130,131], hsa-miR-150 [112,131,132,133], hsa-miR-15a [107,112,134,135,136], hsa-miR-192 [110,131,132,137], hsa-miR-21 [67,107,108,119,138,139,140], hsa-miR-223 [107,112,121,135,141], hsa-miR-29a [112,129,131,142], hsa-miR-375 [112,129,132,143,144,145], and hsa-miR-9 [112,129,145,146]. However, some of the expression directions are not consistent, possibly due to the differences in study population and sample type.

While most studies used univariate analysis to identify biomarkers, some used multivariate analyses to develop classifiers. For example, a logistic regression model with hsa-miR-148b, hsa-miR-223, hsa-miR-130a, and hsa-miR-19a was able to differentiate T2D patients from those with impaired glucose regulation (AUC = 0.90, 95%CI = 0.86–0.94). The same model had a similar performance in a validation set of 87 patients with impaired glucose tolerance and 113 T2DM patients (AUC = 0.88, 95%CI = 0.83–0.94) [141]. Polygenic risk scores were also developed to predict T2D complications, using three [138] or five [146] miRNAs. A study on short-term intensive insulin therapy found that a Random Forest predictor containing baseline hsa-miR-145-5p, hsa-miR-29c-3p, and HbA1c could predict responses to therapy better than models with only clinical parameters [137]. These studies further support that circulating blood miRNAs are promising T2D biomarkers.

**Table 8:** microRNA biomarkers from blood samples for T2D.

Sample Type	Profiling Method	Sample Size (Controls, T2D, Other)	Biomarker	Ref
Endothelial progenitor cells	qRT-PCR	15, 15	hsa-miR-21, hsa-miR-27a, hsa-miR-27b, hsa-miR-126, hsa-miR-130	[108]
PBMC	qRT-PCR	20, 20	hsa-miR-146a	[64]
Plasma	Microarray and qRT-PCR	94, 112, 72 *	hsa-let-7b, hsa-miR-142, hsa-miR-144, hsa-miR-29a	[142]
Plasma	RNA Sequencing	0, 145, 145 *	hsa-miR-122-5p, hsa-miR-210-3p, hsa-miR-3200-3p, hsa-miR-376b-3p, hsa-miR378a-3p, hsa-miR-4532-5p, hsa-miR-483-5p and hsa-miR-660-3p	[125]
Plasma	Microarray and qRT-PCR	50, 50, 50 *	hsa-miR-1249, hsa-miR-320b, hsa-miR-572, hsa-miR-6069	[147]
Plasma	qRT-PCR	30, 30, 30 *	hsa-miR-126	[109]
Plasma	qRT-PCR	20, 20	hsa-miR-126	[113]
Plasma	qRT-PCR	0, 0, 36 $	hsa-miR-126	[123]
Plasma	qRT-PCR	58, 69, 34 #, 124 **	hsa-miR-126-3p	[118]
Plasma	qRT-PCR	107, 76, 117 **	hsa-miR-126-3p, hsa-miR-21-5p	[119]
Plasma	qRT-PCR	30, 30, 30 *	hsa-miR-126-5p and hsa-miR-181b	[62]
Plasma	qRT-PCR	20, 54, 46 **	hsa-miR-126, hsa-miR-210	[111]
Plasma	qRT-PCR	80, 55	hsa-miR-126, hsa-miR-26a	[114]
Plasma	qRT-PCR	35, 30, 10 #, 18 **	hsa-miR-140-5p, hsa-miR-142-3p, hsa-miR-222, hsa-miR-423-5p, hsa-miR-192, hsa-miR-125b, hsa-miR-195, hsa-miR-130b, hsa-miR-532-5p, hsa-miR-126	[110]
Plasma	qRT-PCR	7, 18, 17 $	hsa-miR-140-5p, hsa-miR-222, hsa-miR-142-3p, hsa-miR-192	[110]
Plasma	qRT-PCR	0, 0, 24 $	hsa-miR-145-5p, hsa-miR-29c-3p, hsa-miR-192, hsa-miR-20a, hsa-let-7b, hsa-miR-802, hsa-miR-34a	[137]
Plasma	qRT-PCR	90, 58, 32 **	hsa-miR-146a	[127]
Plasma	qRT-PCR	9, 9, 9 *	hsa-miR-148a-3p, hsa-miR-222-3p, hsa-miR-342-3p, hsa-miR-143-3p, hsa-miR-320b, hsa-miR-320c	[148]
Plasma	qRT-PCR	20, 23, 26 **	hsa-miR-191, hsa-miR-200b	[149]
Plasma	qRT-PCR	50, 50, 50 #, 50 **	hsa-miR-195-5p, hsa-miR-130a-3p	[150]
Plasma	Microarray, qRT-PCR	80, 9, 71 **	hsa-miR-20b, hsa-miR-21, hsa-miR-24, hsa-miR-15a, hsa-miR-126, hsa-miR-191, hsa-miR-197, hsa-miR-223, hsa-miR-320, hsa-miR-486, hsa-miR-28-3p	[107]
Plasma	qRT-PCR	115, 65, 124 **	hsa-miR-21	[151]
Plasma	qRT-PCR	285, 285, 855 **	hsa-miR-21, hsa-miR-218, hsa-miR-211	[138]
Plasma	qRT-PCR	119, 33	hsa-miR-24, hsa-miR-29b, hsa-miR-144	[152]
Plasma	qRT-PCR	20, 91, 95	hsa-miR-29b, hsa-miR-200b	[153]
Plasma	qRT-PCR	355, 107	hsa-miR-30a-5p, hsa-miR-150, hsa-miR-9, hsa-miR-15a, hsa-miR-28-3p, hsa-miR-29a, hsa-miR-103, hsa-miR-223, hsa-miR-126, hsa-miR-145, and hsa-miR-375	[154]
Plasma	qRT-PCR	100, 100	hsa-miR-375	[143]
Plasma	qRT-PCR	0, 0, 40 $	hsa-miR-378, hsa-miR-126-3p, hsa-miR-223-5p	[121]
Plasma and plasma exosome	qRT-PCR	26, 26, 24 *	hsa-miR-15a	[134]
Plasma exosome	qRT-PCR	18, 12	hsa-miR-326, hsa-let-7a, hsa-let-7f	[155]
Platelet	qRT-PCR	46, 43, 48 #, 36 **	hsa-miR-103b	[70]
Serum	RNA sequencing and qRT-PCR	3, 50, 29 **	hsa-let-7a-5p, hsa-miR-novel-chr5_15976, hsa-miR-28-3p, hsa-miR-151a-5p, and hsa-miR-148a-3p	[142]
Serum	qRT-PCR	49, 155	hsa-miR-101, hsa-miR-375, hsa-miR-802	[144]
Serum	qRT-PCR	100, 100, 86 *	hsa-miR-126	[115]
Serum	qRT-PCR	138, 160, 157 *	hsa-miR-126	[116]
Serum	qRT-PCR	40, 40, 40 #, 40 **	hsa-miR-128	[156]
Serum	qRT-PCR	0, 30, 20 **	hsa-miR-1281, hsa-miR-4687-5p, hsa-miR-4688, hsa-miR-1260a, and hsa-miR-766-3p	[157]
Serum	qRT-PCR	49, 49, 47 *	hsa-miR-130b-3p, hsa-miR-374a-5p	[158]
Serum	qRT-PCR	40, 22, 34 **	hsa-miR-146a	[128]
Serum	qRT-PCR	35, 54, 16 *, 28 **	hsa-miR-146a	[130]
Serum	qRT-PCR	68, 215, 178 *	hsa-miR-148b, hsa-miR-223, hsa-miR-130a, and hsa-miR-19a	[141]
Serum	qRT-PCR	138, 136, 254 **	hsa-miR-154-5p	[159]
Serum	RNA Sequencing and qRT-PCR	225, 200, 470 **	hsa-miR-16, hsa-miR-23-3p, hsa-miR-122-5p, hsa-miR-198, hsa-miR-199a-3p, hsa-miR-221, and hsa-miR-34	[126]
Serum	RNA sequencing	0, 11, 10 **	hsa-miR-190a-5p, hsa-miR-4448, hsa-miR-338-3p, hsa-miR-485-5p, and hsa-miR-9-5p	[146]
Serum	Microarray and qRT-PCR	25, 50, 42 **	hsa-miR-20a, hsa-miR-99b, hsa-miR-122-5p, and hsa-miR-486-5p	[124]
Serum	qRT-PCR	81, 30, 50 **	hsa-miR-20b, hsa-miR-17-3p, HOTAIR (lncRNA), and MALAT1 (lncRNA)	[160]
Serum	qRT-PCR	42, 45	hsa-miR-21	[139]
Serum	qRT-PCR	33, 37, 64 **	hsa-miR-221	[161]
Serum	Nanostring and qRT-PCR	0, 24, 18 *	hsa-miR-298, hsa-miR-491-5p, hsa-miR-1307-3p	[162]
Serum	qRT-PCR	0, 45, 45 **	hsa-miR-3197 and hsa-miR-2116-5p	[163]
Serum	qRT-PCR	50, 50, 50 #, 50 **	hsa-miR-342 and hsa-miR-450	[164]
Serum	qRT-PCR	50, 27, 23 **	hsa-miR-421, hsa-miR-212-5p, hsa-miR-3909, hsa-miR-4677-3p, and hsa-miR-4766-5p	[165]
Serum	qRT-PCR	20, 13, 20 #, 16 **	hsa-miR-503	[166]
Serum	qRT-PCR	92, 92, 92 **	hsa-miR-571, hsa-miR-661, hsa-miR-770-5p, hsa-miR-892b, hsa-miR-1303	[167]
Serum	qRT-PCR	25, 25, 25 #, 25 **	hsa-miR-593	[168]
Serum	qRT-PCR	19, 18, 19 *	hsa-miR-9, hsa-miR-29a, hsa-miR-30d, hsa-miR-34a, hsa-miR-124a, hsa-miR-146a, and hsa-miR-375	[129]
Serum	qRT-PCR	5, 10	hsa-miR-455-5p, hsa-miR-454-3p, hsa-miR-144-3p, hsa-miR-96-5p, hsa-miR-665 and hsa-miR-766-3p	[169]
Serum and serum exosomes	qRT-PCR	74, 76, 76 **	hsa-miR-7	[170]
Serum exosomes	qRT-PCR	24, 14, 17 *	hsa-miR-10b, hsa-miR-194, hsa-miR-223-3p, hsa-miR-15a, hsa-miR-93	[135]
Serum exosomes	qRT-PCR	20, 21	hsa-miR-20b-5p and hsa-miR-150-5p	[133]
Serum exosomes	Microarray and qRT-PCR	0, 20, 24 **	hsa-miR-377-3p	[67]
Whole blood	qRT-PCR	62, 104, 108 **	hsa-let-7a-2	[171]
Whole blood	qRT-PCR	30, 30, 30 *	hsa-miR-122, hsa-miR-126-5p, hsa-miR-146a	[120]
Whole blood	qRT-PCR	45, 45, 45 **	hsa-miR-126	[117]
Whole blood	RNA Sequencing	3, 3	hsa-miR-1271-5p, hsa-miR-130a-3p, hsa-miR-130b-3p, andhsa-miR-574-3p	[172]
Whole blood	qRT-PCR	972, 94, 207 *	hsa-miR-1299, hsa-miR-126-3p, hsa-miR-30e-3p	[122]
Whole blood	qRT-PCR	8, 13, 8 *	hsa-miR-144, hsa-miR-146a, hsa-miR-150, hsa-miR-182, hsa-miR-192, hsa-miR-30d, hsa-miR-29a, hsa-miR-320	[131]
Whole blood	qRT-PCR	24, 24, 22 *	hsa-miR-15a	[136]
Whole blood	qRT-PCR	30, 30, 30 *	hsa-miR-375, hsa-miR-9	[145]
Whole blood	RNA sequencing and qRT-PCR	4, 4, 4 *	hsa-miR-98-5p, hsa-miR-143-3p, hsa-miR-21-3p, hsa-miR-379-5p	[140]
Whole blood and exosome	qRT-PCR	46, 50	hsa-miR-150, hsa-miR-192,hsa-miR-27a, hsa-miR-320a and hsa-miR-375	[132]

* Pre-diabetes; ** T2D patients with complications or comorbidities; $ T2D patients with treatment; # non-T2D subjects with comorbidities.

### 4.2. Urine

#### 4.2.1. DNA Methylation

The discovery of biomarkers in DNA methylation using urine samples in diabetes is currently an interesting topic of research. Cell type-specific DNA methylation patterns have been widely studied and used to calculate proportions of particular cell types related to diabetes in blood but not in urine [173]. Studies focusing on biomarkers for the diagnosis of T2D have not been validated in a clinical environment. Instead, the researchers have been more interested in developing biomarkers for one specific comorbidity, kidney disease (Table 9) [173,174,175]. Because urine examinations identify changes in gene expression in urine-derived cells [176], they may be helpful for the noninvasive assessment of kidney condition and prediction in early T2D [177].

#### 4.2.2. microRNA

T2D urine miRNA studies mainly studied T2D patients with kidney complications [178,179,180,181,182,183,184], except one study compared patients with different T2D treatments [185] (Table 10). Most studies profiled miRNAs from urinary exosomes, while a few profiled urine supernatants [178,184]. There is little overlap between the reported miRNAs because some studies selected miRNAs based on their study population. Nevertheless, most miRNAs were upregulated in patients with T2D complications than patients without complications. The results suggest that urine could be an interesting non-invasive source of T2D biomarkers, but more studies are needed to validate these findings.

### 4.3. Other Non-Invasive Biomarkers

#### 4.3.1. DNA Methylation

DNA methylation studies using saliva samples have been more frequent in recent years. Despite this, DNA obtained from saliva is a combination of bacterial and human DNA. Hence, there are unique challenges in using DNA from saliva samples in methylation studies that can affect data quality in epidemiological studies, such as T2D [186]. Currently, there are no empirical studies with evidence for DNA methylation in salivary tissue as potential diagnostic biomarkers of T2D. Nonetheless, some studies support the use of DNA methylation from saliva as a prognostic biomarker in comorbidities. In 2011, 39 genes were identified as differentially methylated in two CpG sites in each gene in a group of end-stage renal disease versus diabetes patients without nephropathy. These sites have been involved in kidney development or diabetic nephropathy. As a result, these sites identified in DNA extracted from saliva give proof that they can be used as predictive biomarkers of disease susceptibility [187]. In 2020 a study provided empirical evidence supporting DNA methylation in salivary tissue as a potential predictor of childhood obesity, making *NFR1* a target for further exploration as a biomarker [188].

#### 4.3.2. microRNA

There is only one salivary miRNA study for T2D biomarkers. The study evaluated four miRNAs that have been reported in T2D and periodontal diseases: hsa-mir-146a, hsa-mir-146b, hsa-mir-155, and hsa-mir-203 [189]. These miRNAs were elevated in T2D patients with healthy periodontium compared to healthy controls, with fold changes from 4.9 to 16.5, and were able to distinguish groups with high sensitivity and accuracy (0.75–1 and 0.78–0.85, respectively). The only miRNA that was significantly reduced in T2D patients with periodontitis was hsa-miR-146a, which could predict periodontitis among T2D patients with an accuracy of 0.86. So, salivary miRNAs are promising T2D biomarkers, and more studies are needed to identify more non-invasive biomarkers.

## 5. Conclusions and Future Directions

Table 11 summarizes and classifies candidate omics biomarkers reported in at least two studies. Most of these biomarkers are risk, diagnostic, prognostic, and response biomarkers profiled from blood samples. To the best of our knowledge, no monitoring DNA or RNA biomarkers have been reported for T2D. So, more longitudinal studies are needed to identify monitoring biomarkers and novel risk biomarkers. Despite many biomarkers reported, there is little overlap between studies due to study methodology differences. For genomic studies, the PRSs can potentially improve biomedical outcomes via precision medicine on T2D. However, large-scale biobanks with diverse ethnic populations need to be considered in more studies to address the need for a broad genomic approach to this global disease.

Biomarker overlap is mainly seen in DNA methylation and miRNA studies (Table 11). Twelve genes were reported in at least two studies for genomics studies and were used as risk biomarkers (Table 11). Only three diagnostic and prognostic biomarkers were reported in at least two mRNA studies (Table 11). The reason is that most mRNA T2D studies are targeted, with researchers focusing on previously reported T2D-related genes. In contrast, most genomics and epigenomics studies used untargeted approaches. Future T2D studies should consider untargeted approaches to identify novel biomarkers.

For circulating miRNA studies, 21 diagnostic miRNAs were reported in at least two studies (Table 11). According to DIANA-miRPath (v3.0) [190] and DIANA-TarBase (v7.0) [191], these miRNAs target 108 genes within the insulin signaling pathway (KEGG pathway hsa04910) (Figure S1, Table S2). Some miRNAs target only one gene, such as hsa-miR-28-3p and *SOS2*, while other miRNAs target up to 44 genes within the pathway (Table S2). Although not all miRNA-gene interactions in TarBase were reported from diabetes-related tissues, the finding suggests that diagnostic miRNAs play important roles within the insulin signaling pathway (Figure S1).

Lastly, more research on non-invasive biomarkers is needed, especially on T2D patients with no complications. Non-invasive biomarkers would be better for screening and diagnosing T2D in many people. Validation studies are also necessary to confirm the utility of these biomarkers. Nevertheless, these studies have identified potential T2D biomarkers that could help guide future T2D studies.

## Figures and Tables

**Table 1:** Definitions of biomarkers and potential applications.

Type of Biomarker	BEST Definition	Application/Example
Susceptibility/risk	A biomarker that indicates the potential for developing a disease or medical condition in an individual who does not currently have clinically apparent disease or medical condition.	BRCA1/2 mutations can be used to identify individuals with a predisposition to develop breast cancer
Diagnostic	A biomarker to detect or confirm the presence of a disease or condition of interest or to identify individuals with a subtype of the disease.	HbA1c can be used to identify patients with T2DM
Monitoring	A biomarker measured repeatedly for assessing disease status or medical condition or for evidence of exposure to (or effect of) a medical product or an environmental agent.	Hepatitis C virus or HIV RNA may be measured repeatedly to monitor treatment response
Prognostic	A biomarker to identify likelihood of a clinical event, disease recurrence, or progression in patients who have the disease or medical condition of interest	BRCA1/2 mutations can evaluate the likelihood of second breast cancer.
Predictive	A biomarker used to identify individuals who are more likely than similar individuals without the biomarker to experience a favorable or unfavorable effect from exposure to a medical product or an environmental agent.	BRCA1/2 mutations can identify ovarian cancer patients likely to respond to PARP inhibitors.
Pharmacodynamic/response	A biomarker used to show that a biological response has occurred in an individual who has been exposed to a medical product or an environmental agent.	HbA1c may be used to assess diabetes control after treatment
Safety	A biomarker measured before or after an exposure to a medical product or an environmental agent to indicate the likelihood, presence, or extent of toxicity as an adverse effect.	Neutrophil count can be used to adjust dose for patients on cytotoxic chemotherapy.

**Table 3:** Genomics biomarkers from urine samples for T2D.

Sample Type	Profiling Method	Sample Size (Controls, T2D, Other)	Biomarker	Ref
Blood (plasma or serum) and urine	qRT-PCR	4668, 0, 2290 **	Urine albumin-to-creatinine ratio and *WFS1* (rs10010131)	[41]
Blood (plasma) and urine	qRT-PCR	0, 290, 285 **	Urine albumin and *PGC-1α* (rs8192678)	[42]
Blood and urine	qRT-PCR	35, 0, 42 **	Urine creatinine ratio with mtDNA	[40]

** T2D patients with complications or comorbidities.

**Table 9:** DNA methylation biomarkers from urine samples for T2D.

Sample Type	Profiling Method	Sample Size (Controls, T2D, Other)	Biomarker	Ref
Urine	Global DNA Methylation ELISA kit	0, 0, 308 **	5-methyl-2′-deoxycytidine (*5MedC*)	[174]
Urine	Illumina Infinium MethylationEPIC Kit	9, 0, 4 **	*SMTNL2* and *G6PC*	[173]

** T2D patients with complications or comorbidities.

**Table 10:** microRNA biomarkers from urine samples for T2D.

Sample Type	Profiling Method	Sample Size (Controls, T2D, Other)	Biomarker	Ref
Urine	qRT-PCR	85, 86, 92 **	hsa-miR-126	[184]
Urine	qRT-PCR	0, 41, 42 **	hsa-miR-29a	[178]
Urinary exosome	qRT-PCR	54, 56, 110 **	hsa-miR-133b, hsa-miR-342, hsa-miR-30a	[182]
Urinary exosome	qRT-PCR	40, 40, 80 **	hsa-miR-15b-5p, hsa-let-7i-5p, hsa-miR-135b-5p, hsa-miR-24-3p, hsa-miR-27b-3p, hsa-miR-30a-5p, hsa-miR-197-3p	[179]
Urinary exosome	qRT-PCR	44, 46, 90 **	hsa-miR-15b, hsa-miR-34a and hsa-miR-636	[181]
Urinary exosome	qRT-PCR	10, 30, 50 **	hsa-miR-192, hsa-miR-194, and hsa-miR-215	[180]
Urinary exosome	Nanostring and qPCR	7, 23, 34 **	hsa-miR-23a-3p	[185]
Urinary exosome	Microarray and qRT-PCR	0, 14, 14 **	hsa-miR-4687-3p, hsa-miR-4534, hsa-miR-5007-3p	[183]

** T2D patients with complications or comorbidities.

**Table 11:** Classification of potential genomics, transcriptomics, and epigenomics biomarkers of T2D.

Biomarker Type	Definition	Genomics (SNPs)	Transcriptomics (mRNA)	DNA Methylation	miRNA
Risk	Risk for developing T2D in those who appear healthy	*PPARG*, *FTO*, *CDC123*, *TCF7L2*, *CDKAL1*, *WFS1*, *KCNJ11*, *SLC30A8*, *ADAMTS9*, *IGF2BP2*, *TSPAN8*, *JAZF1*	-	*ABCG1*, *PHOSPHO1*, *SOCS3*, *SREBF1*, *TXNIP*	-
Diagnostic	Confirming the presence of T2D or identifying a subset of T2D	*Ruminococcaceae* (Gut microbiome)	*IL-6*, *IL-8*, *TTP*	-	hsa-miR-126, hsa-miR-126-3p, hsa-miR-126-5p, hsa-miR-122-5p, hsa-miR-144, hsa-miR-146a, hsa-miR-150, hsa-miR-15a, hsa-miR-191, hsa-miR-192, hsa-miR-20b, hsa-miR-21, hsa-miR-221, hsa-miR-223, hsa-miR-24, hsa-miR-27a, hsa-miR-28-3p, hsa-miR-29a, hsa-miR-29b, hsa-miR-30d, hsa-miR-34a, hsa-miR-375
Monitoring	Measured repeatedly to assess T2D status	-	-	-	-
Prognostic	To identify the likelihood of progressing from IGT to T2D or developing T2D complications	-	*IL-6*, *IL-8*, *TTP*	-	hsa-miR-122-5p, hsa-miR-126, hsa-miR-126-3p, hsa-miR-143-3p, hsa-miR-144, hsa-miR-146a, hsa-miR-192, hsa-miR-194, hsa-miR-21, hsa-miR-29a, hsa-miR-320b, hsa-miR-34a, hsa-miR-375
Predictive	To identify individuals who will experience favorable/unfavorable medical response, compared to those without the biomarker	-	-	-	-
Pharmacodynamic/response	To observe T2D treatment response, not measured repeatedly	-	*SOD1*	-	hsa-miR-126, hsa-miR-192
Safety	To evaluate toxicity response to a treatment	-	-	-	-

## Supplementary Materials

The following are available online at https://www.mdpi.com/article/10.3390/ijms23010295/s1.
