# Supplementary material for: Circulating Nucleic Acid-Based Biomarkers of Type 2 Diabetes
Source: Int J Mol Sci. 2021 Dec 28;23(1):295. doi: 10.3390/ijms23010295 (PMC8745431; doi:10.3390/ijms23010295)
Supplement: Supplementary file 1 [file ijms-23-00295-s001.zip › ijms-1525985-supplementary.pdf]

Table S1. List of gene symbols and corresponding gene names.

Gene	Name
ABCG1	ATP Binding Cassette Subfamily G Member 1
ADAM30	ADAM Metallopeptidase Domain 30
ADAMTS9	ADAM Metallopeptidase With Thrombospondin Type 1 Motif 9
AEBP1	AE Binding Protein 1
BCL11A	B-cell lymphoma/leukemia 11A
BSP	Bone sialoprotein
C2CD4B	C2 Calcium Dependent Domain Containing 4B
CCDC57	Coiled-Coil Domain Containing 57
CD24	CD24 Molecule
CDC123	Cell Division Cycle 123
CDKAL1	CDK5 Regulatory Subunit Associated Protein 1 Like 1
CDKN2A	Cyclin Dependent Kinase Inhibitor 2A
CDKN2B	Cyclin Dependent Kinase Inhibitor 2B
CDKNA	Cyclin Dependent Kinase Inhibitor A
CHGA	Chromogranin A
CPT1A	Carnitine Palmitoyltransferase 1A
CXXC4	CXXC Finger Protein 4
CYP2C9	Cytochrome P450 family 2 subfamily C member 9
DCD	Dermcidin
EGFR	Epidermal Growth Factor Receptor
ELOVL5	ELOVL Fatty Acid Elongase 5
ERRγ	Estrogen-related receptor γ
FTO	FTO Alpha-Ketoglutarate Dependent Dioxygenase
G6PC	Glucose-6-phosphatase catalytic subunit 1
G6PD	Glucose-6-phosphate dehydrogenase deficiency
GAPDH	Glyceraldehyde-3-Phosphate Dehydrogenase
GLB1	Galactosidase Beta 1
HHEX	Haematopoietically Expressed Homeobox
IDE	Insulin Degrading Enzyme
IFN-g	Interferon gamma
IGF2BP2	Insulin Like Growth Factor 2 MRNA Binding Protein 2
IGFBP-1	Insulin-like growth factor-binding protein 1
IL-23	Interleukin 23
IL-6	Interleukin 6
IL-8	Interleukin-8
INS	Insulin
JAZF1	JAZF Zinc Finger 1
KCNJ11	Potassium Inwardly Rectifying Channel Subfamily J Member 11
KCNK11	Potassium Two Pore Domain Channel Subfamily K Member 11
KRAS	KRAS Proto-Oncogene, GTPase
LINE-1	Long Interspersed Nucleotide Element 1
LOXL2	Lysyl Oxidase Like 2
LRRK2	Leucine Rich Repeat Kinase 2
MSI2	Musashi RNA Binding Protein 2
MUC19	Mucin 19
NDUFB2	NADH:Ubiquinone Oxidoreductase Subunit B2
NFE2L2	NFE2 Like BZIP Transcription Factor 2
NF-kB	Nuclear Factor kappa-light-chain-enhancer of activated B cells
NFR1	Nuclear Respiratory Factor 1

NOD-2	Nucleotide-binding oligomerization domain 2
NOTCH2	Notch Receptor 2
NPHS1	Nephrin
NPHS2	Podocin
OAZ1	Ornithine Decarboxylase Antizyme 1
PEG3	Paternally Expressed 3
PGC-1α	Peroxisome proliferator-activated receptor gamma coactivator 1-alpha
PHOSPHO1	Phosphoethanolamine/Phosphocholine Phosphatase 1
PODXL	Podocalyxin
PPARG / PPAR-γ	Peroxisome Proliferator Activated Receptor Gamma
PPARGC1A	PPARG Coactivator 1 Alpha
PRKCZ	Protein Kinase C Zeta
PSMB2	Proteasome 20S Subunit Beta 2
PSMD6	Proteasome 26S Subunit, Non-ATPase 6
SAMD12	Sterile Alpha Motif Domain Containing 12
SAT1	Spermidine/Spermine N1-Acetyltransferase 1
SFRP4	Secreted Frizzled Related Protein 4
SHBG	Sex hormone binding globulin
SLC12A1	Solute Carrier Family 12 Member 1
SLC13A2	Solute Carrier Family 13 Member 2
SLC1A5	Solute Carrier Family 1 Member 5
SLC30A8	Solute Carrier Family 30 Member 8
SLC44A3	Solute Carrier Family 44 Member 3
SMAD1	SMAD Family Member 1
SMTNL2	Smoothelin Like 2
SOCS3	Suppressor Of Cytokine Signaling 3
SOD1	Superoxide Dismutase 1
SOS2	SOS Ras/Rho Guanine Nucleotide Exchange Factor 2
SREBF1	Sterol Regulatory Element Binding Transcription Factor 1
TCF2	transcription factor-2
TCF7L2	Transcription factor 7-like 2
TFAM	Transcription Factor A, Mitochondrial
THADA	Thyroid adenoma-associated
TLR2	Toll-like receptor 2
TLR4	Toll Like Receptor 4
TMEM72	Transmembrane Protein 72
TNF-a	Tumor necrosis factor α
TPM4	Tropomyosin 4
TRAF-6	Tumor necrosis factor receptor associated factor 6
TSPAN8	Tetraspanin 8
TTP	Tristetraproline
TXNIP	Thioredoxin Interacting Protein
UGT1A1	UDP glucuronosyltransferase 1 family, polypeptide A1
UMOD	Uromodulin
VEGF	Vascular endothelial growth factor
VEGFA	Vascular Endothelial Growth Factor A
WFS1	Wolframin ER Transmembrane Glycoprotein

Table S2. List of miRNA-gene interactions between diagnostic miRNAs and Insulin Signaling Pathway genes, according to DIANA-miRPath (v3.0) and DIANA-TarBase (v7.0).

	miRNA	Number of interactions	Genes
1	hsa-miR-126-5p	16	*AKT1, CRK, GRB2, GSK3B, IRS1, IRS2, KRAS, MAPK1, PDPK1, PIK3CA, PIK3CD, PIK3R2, PRKAA1, PRKAB2, PTPN1, RPS6KB1*
2	hsa-miR-126-3p	8	*AKT1, CRK, IRS1, IRS2, MAPK1, PIK3CA, PIK3CD, PIK3R2*
3	hsa-miR-122-5p	27	*ACACA, ACACB, AKT1, CBL, EXOC7, G6PC3, GYS1, INSR, MKNK1, MKNK2, MTOR, PDPK1, PHKA1, PHKA2, PIK3CD, PIK3CG, PPP1R3A, PPP1R3E, PRKAA1, PRKAB2, PRKAG2, PRKAR1A, PRKAR2A, RAF1, RPS6KB1, SOS2, TSC1*
4	hsa-miR-144-3p	2	*MKNK2, MTOR*
5	hsa-miR-146a-5p	4	*AKT2, GSK3B, PRKAA1, RPS6*
6	hsa-miR-150	5	*CALM2, EIF4E2, MAP2K1, MKNK2, PRKACB*
7	hsa-miR-15a	38	*ACACA, AKT1, AKT2, AKT3, BRAF, CALM1, CALM3, CBL, CRK, CRKL, EXOC7, FASN, FLOT2, G6PC3, GRB2, HK1, IKBKB, INPPL1, IRS4, KRAS, MAP2K1, MAPK8, MAPK9, MKNK2, PHKA1, PHKG2, PIK3CA, PPP1CA, PPP1CC, PRKAB2, PRKACA, PRKAG1, PRKAR1A, PRKAR2A, PTPRF, RAPGEF1, SHC1, TSC1*
8	hsa-miR-191-3p	7	*CRK, CRKL, MKNK2, PPP1CB, PRKACA, RAPGEF1, SOCS4*
9	hsa-miR-192-3p	13	*AKT3, CRK, IRS4, MAPK9, MKNK2, PPP1CA, PPP1CB, PPP1CC, PRKAA2, PRKAR1A, PTPN1, RPS6KB1, SREBF1*
10	hsa-miR-20b	15	*RAPGEF1, CRK, MAPK9, TRIP10, PIK3R3, SOCS1, MAPK8, PIK3R1, PRKAB2, PRKAR1A, AKT3, PPP1R3B, MKNK2, MAPK1, PRKACB*
11	hsa-miR-21	24	*CALM1, CRK, CRKL, FASN, HK2, INPP5K, MAPK1, MKNK2, MTOR, NRAS, PIK3R1, PIK3R3, PPP1CC, PPP1R3B, PRKAA2, PRKAB2, PRKAG1, PTPRF, RAPGEF1, RHOQ, SOCS4, SOS2, TSC1, TSC2*
12	hsa-miR-221	15	*ACACA, CRKL, FOXO1, MAP2K1, MAPK3, MKNK2, MTOR, PRKAA2, PRKAB2, PRKAR1A, PRKCZ, PTPN1, PTPRF, RAF1, RPTOR*
13	hsa-miR-223	4	*CBL, CRK, MTOR, PIK3CD*
14	hsa-miR-24	26	*FASN, GSK3B, TSC1, CBL, INPPL1, CALM1, PIK3CB, FLOT1, PCK2, PDE3A, RAF1, PRKAR1B, PTPRF, TSC2, PIK3R3, IRS4, IRS1, PRKAB2, EIF4E2, PYGB, PDPK1, MKNK2, EXOC7, RPS6KB1, PPP1R3D, ACACA*
15	hsa-miR-27a	44	*ACACA, AKT1, BRAF, CALM2, CALM3, CBL, CBLB, CRK, EIF4EBP1, FASN, FOXO1, G6PC3, GRB2, GSK3B, IRS1, MAP2K2, MAPK1, MAPK3, MAPK8, MKNK1, MKNK2, MTOR, NRAS, PDPK1, PIK3CB, PIK3R1, PIK3R3, PPARGC1A, PPP1CA, PPP1CC, PRKAA1, PRKAG1, PRKAR1A, PRKAR2A, PRKCI, PRKX, PTPN1, PYGL, RHEB, RPS6, RPS6KB1, SHC1, SREBF1, TSC1*
16	hsa-miR-28-3p	1	*SOS2*
17	hsa-miR-29a	24	*ACACA, CALM3, CRKL, EIF4E2, FASN, GRB2, GSK3B, MAP2K2, MAPK8, NRAS, PDE3A, PIK3R1, PIK3R3, PPP1CC, PPP1R3C, PRKAA1, PRKACB, PRKAG1, PTPRF, RHEB, SHC1, SOCS4, SOS1, SREBF1*
18	hsa-miR-29b	15	*FASN, GSK3B, NRAS, CALM3, PDE3A, PTPRF, SREBF1, PIK3R3, MAPK8, AKT1, PIK3R1, SOS1, PRKAA1, EIF4E2, ACACA*
19	hsa-miR-30d-3p	23	*CALM1, FASN, GRB2, INSR, IRS2, MAPK8, MKNK1, MKNK2, NRAS, PHKA2, PIK3CA, PPARGC1A, PPP1CB, PPP1CC, PRKAA1, PRKAB2, PRKAG2, PRKAR1A, PRKAR2A, PTPN1, RPS6KB1, SOCS1, SOCS3*
20	hsa-miR-34a	44	*ACACA, AKT2, ARAF, BAD, BRAF, CALM1, CBLC, CRK, CRKL, EIF4E2, EIF4EBP1, ELK1, FASN, FLOT1, FLOT2, GYS1, HK1, INPP5K, MAP2K1, MAP2K2, MAPK1, MAPK8, MKNK2, PCK2, PHKA1, PHKA2, PIK3CA, PIK3R2, PKLR, PPP1CA, PPP1CC, PPP1R3E, PRKACA, PRKACB,*

			PRKAR1A, PRKAR2B, PRKX, PYGB, RAF1, RHEB, SOCS2, SOCS4, SORBS1, TSC2
21	hsa-miR-375	11	*RAPGEF1, PRKAA2, CALM3, RAF1, PTPN1, PTPRF, RPS6, HK2, PRKX, IRS1, RHOQ*

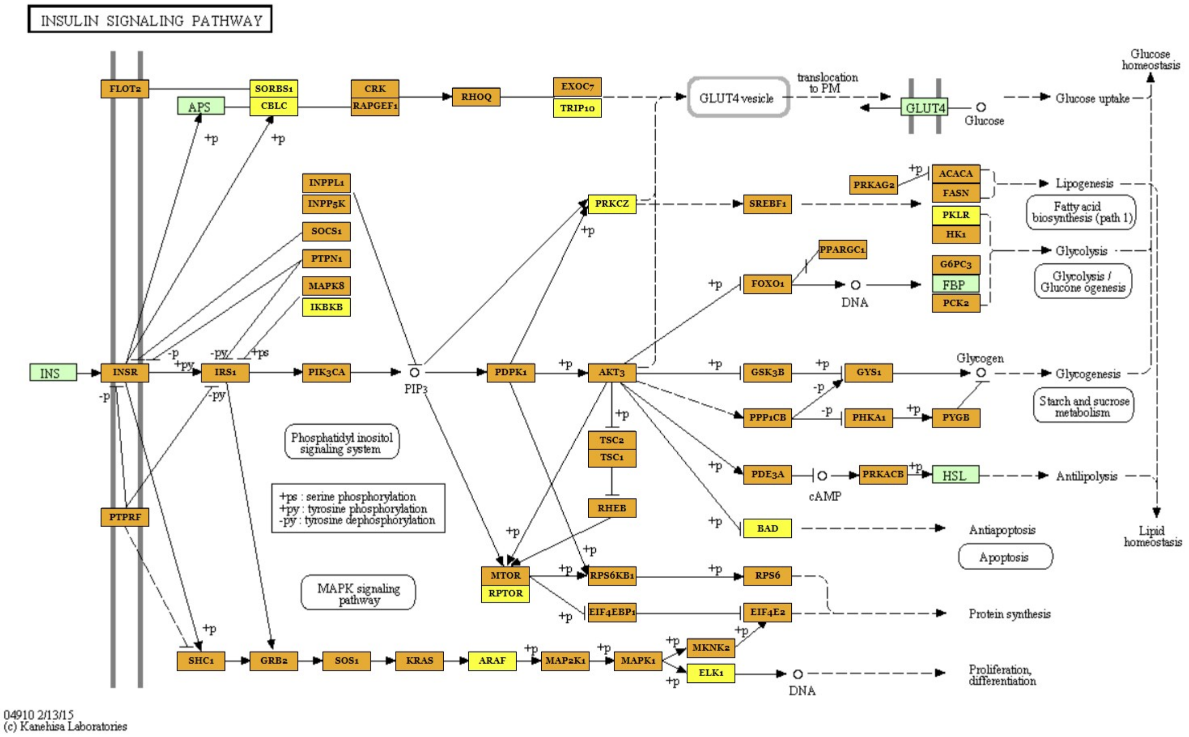

Figure S1. Diagram of genes targeted by diagnostic miRNAs (Table 11) within the Insulin Signaling Pathway, according to DIANA-miRPath (v3.0) and DIANA-TarBase (v7.0). Color labels indicate the number of miRNA-gene interactions: yellow, only one miRNA-gene interaction; orange, more than one miRNAs target the gene; green, no reported miRNA-gene interaction in the database. .
